# Non-HIV Immunocompetent Patient with COVID-19 and Severe *Pneumocystis jirovecii* Pneumonia Co-Infection

**DOI:** 10.3201/eid3009.240615

**Published:** 2024-09

**Authors:** Songsong Yu, Tiecheng Yang

**Affiliations:** Beijing Shijitan Hospital, Capital Medical University, Beijing, China

**Keywords:** COVID-19, 2019 novel coronavirus disease, coronavirus disease, severe acute respiratory syndrome coronavirus 2, SARS-CoV-2, viruses, fungi, respiratory infections, zoonoses, co-infection, Pneumocystis jirovecii pneumonia

## Abstract

*Pneumocystis jirovecii* pneumonia is an opportunistic infection that affects HIV-infected and immunocompromised persons and rarely affects immunocompetent patients. However, after the advent of the COVID-19 pandemic, some COVID-19 patients without immunocompromise or HIV were infected with *P. jirovecii*. Clinical manifestations were atypical, easily misdiagnosed, and rapidly progressive, and the prognosis was poor.

*Pneumocystis jirovecii* pneumonia (PJP) is an opportunistic infection that usually affects immunocompromised patients, most commonly those with HIV infection (*1*). PJP in immunocompetent persons is extremely rare (*2*) particularly among middle-aged persons. Currently, the number of non-HIV patients with PJP is rapidly increasing because of organ transplantation, improvements in diagnosis and treatment of autoimmune diseases, and use of immunosuppressive drugs (including corticosteroids or immunomodulatory monoclonal antibodies). The mortality rate for patients with PJP but not HIV is significantly higher than for those with HIV (up to 30%) (*3*).

Since 2019, COVID-19 infection has become a threat to human health, damaging the epithelial barrier of the airway and inducing abnormal immune responses, which in turn leads to deregulation of the immune system. Therefore, ≈19% of patients with COVID-19 experience pulmonary coinfections (*4*). We report the case of an immunocompetent woman in China who was co-infected with COVID-19 and *P. jirovecii.*

## The Case

**Figure 1:**
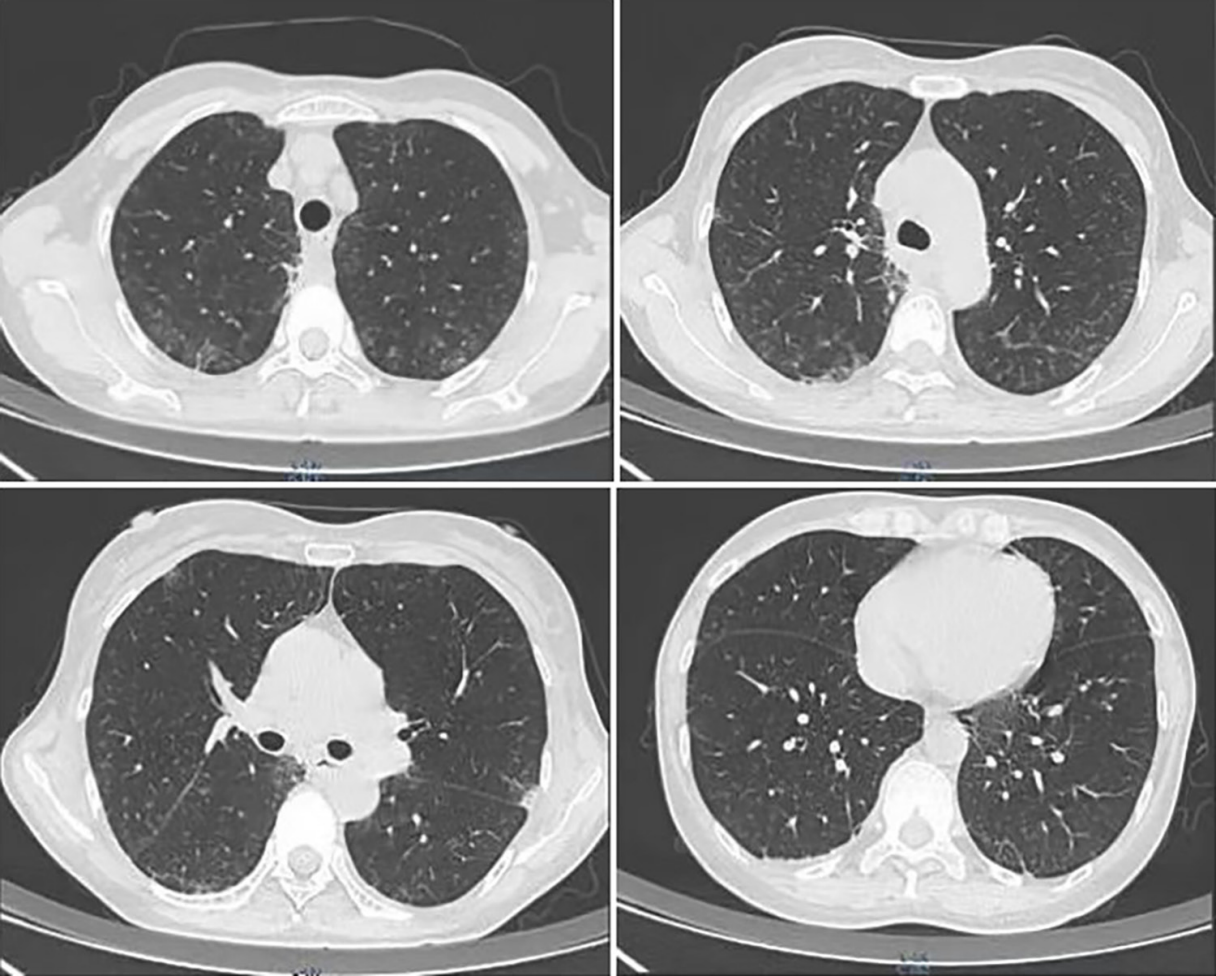
Chest computed tomography scans of immunocompetent patient in China who was co-infected with COVID-19 and non-HIV severe *Pneumocystis jirovecii* pneumonia, performed on June 22, 2023. Images show bronchial vascular bundles blurred in bilateral lungs with multiple tree-in-bud signs, multiple ground-glass shadows, and bilateral subpleural consolidation.

**Figure 2:**
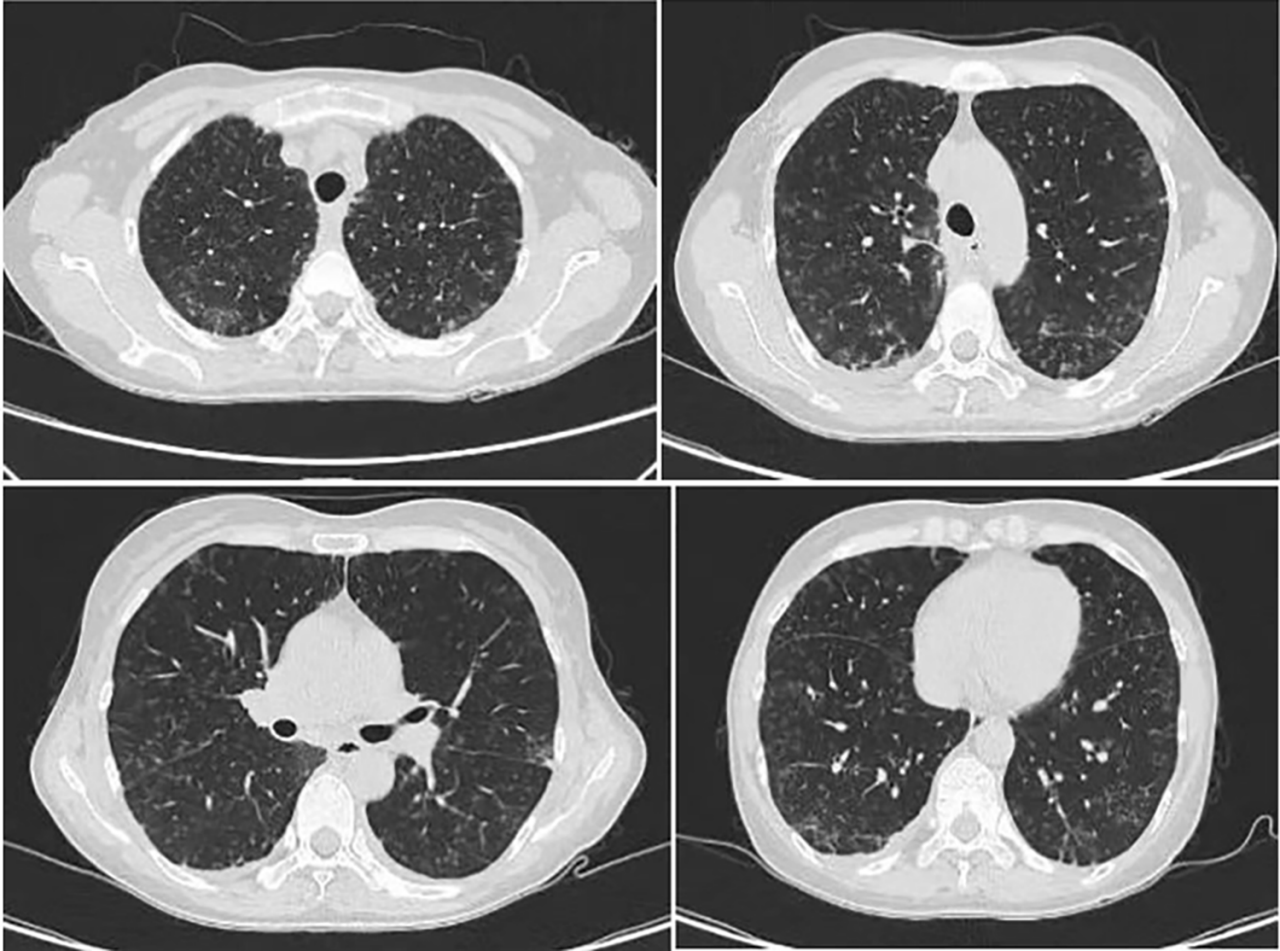
Chest computed tomography scans of immunocompetent patient in China who was co-infected with COVID-19 and non-HIV severe *Pneumocystis jirovecii* pneumonia, performed on June 25, 2023. Images show multiple tree-in-bud signs, ground-glass shadows, and substantially increased bilateral subpleural consolidation in the lungs.

On June 25, 2023, a 52-year-old woman visited the emergency department of Beijing Shijitan Hospital, Capital Medical University (Beijing, China), with an 8-day history of fever (maximum 39.5°C) accompanied by weakness and anorexia and a 5-day history of cough with expectoration. Until 5 days before admission, she had not experienced coughing, expectoration, chills, vomiting, sore throat, or shortness of breath. Results of influenza A/B virus antigen and COVID-19 testing were negative. Laboratory findings were peripheral leukocyte count 5.25 × 10^9^ cells/L (85.5% neutrophils, 8.9% lymphocytes) and C-reactive protein level 118.03 mg/L. Chest computed tomography (CT) scan indicated blurred bronchial vascular bundles in bilateral lungs with multiple tree-in-bud signs, multiple ground-glass shadows, and subpleural consolidation (Figure 1). After receiving intravenous moxifloxacin (0.4 g/d for 3 days), the patient still had high fever, and chest CT scan showed substantially increased bilateral tree-in-bud signs, ground-glass shadows, and subpleural consolidation in lungs (Figure 2). She had no relevant medical history.

At admission, physical examination revealed rough breathing sounds. Peripheral leukocyte count was 4.35 × 10^9^ cells/L (89.1% neutrophils, 8.9% lymphocytes, 1.6% monocytes), and C-reactive protein level was 199.96 mg/L. Serum alanine aminotransferase was 61 U/L, aspartate aminotransferase 65 U/L, lactate dehydrogenase 283 U/L, albumin 33.6 g/L, and plasma D-dimer 2,902 ng/mL. Arterial blood gas indicated a pH of 7.46, partial pressure of CO_2_ (pCo_2_) 35 mm Hg, and partial pressure of O_2_ (pO_2_) 78 mm Hg (fraction of inspired O_2_ [FiO_2_] 29%). Results were negative for serum mycobacterium tuberculosis antibody, Epstein-Barr virus capsid IgM, cytomegalovirus IgM, galactomannan and 1,3-β-d-glucan, serum IgE, antinuclear antibody, antineutrophilic cytoplasmic antibody, and tumor markers. Blood and sputum culture results were unremarkable.

**Figure 3:**
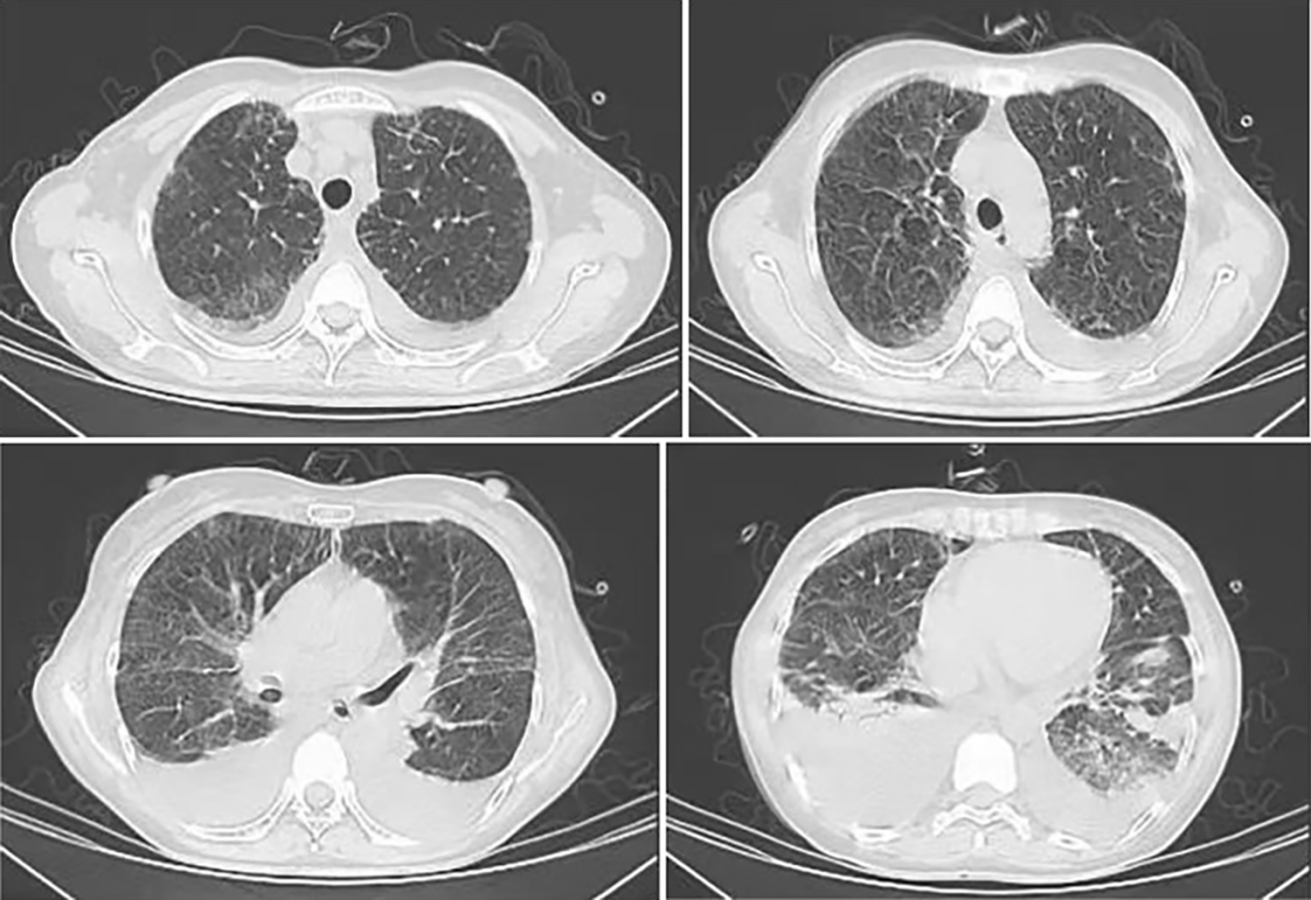
Chest computed tomography scans of immunocompetent patient in China who was co-infected with COVID-19 and non-HIV severe *Pneumocystis jirovecii* pneumonia, performed on June 30, 2023. Images show blurred bronchovascular bundles, interlobular thickening, and subpleural small-band of ground-glass changes in bilateral lungs, with increased bilateral pleural effusion.

After admission, the patient received moxifloxacin and piperacillin/tazobactam, but her fever remained (maximum 40.7°C), accompanied by loss of appetite, aggravated weakness, and shortness of breath after activity. On hospitalization day 7, arterial blood gas indicated pH 7.45, pCO_2_ 46 mm Hg, and pO_2_ 59 mm Hg (FiO_2_ 41%). Chest CT scan showed bilateral blurred bronchovascular bundles, interlobular thickening, and subpleural small-band ground-glass changes in the lungs with increased bilateral pleural effusion (Figure 3).

Culture of bronchalveolar lavage fluid (BALF) from the basal segment of the lower left lung revealed no bacteria and fungi. However, metagenomic next-generation sequencing (mNGS) of BALF detected *P. jirovecii* (222 sequences), *Candida albicans* (2,393 sequences), and SARS-CoV-2 (37 sequences). Peripheral T-lymphocyte subsets included 80/μL CD4+ and 196/μL CD8+ T cells. The diagnosis was COVID-19 with severe *P. jirovecii* co-infection.

**Figure 4:**
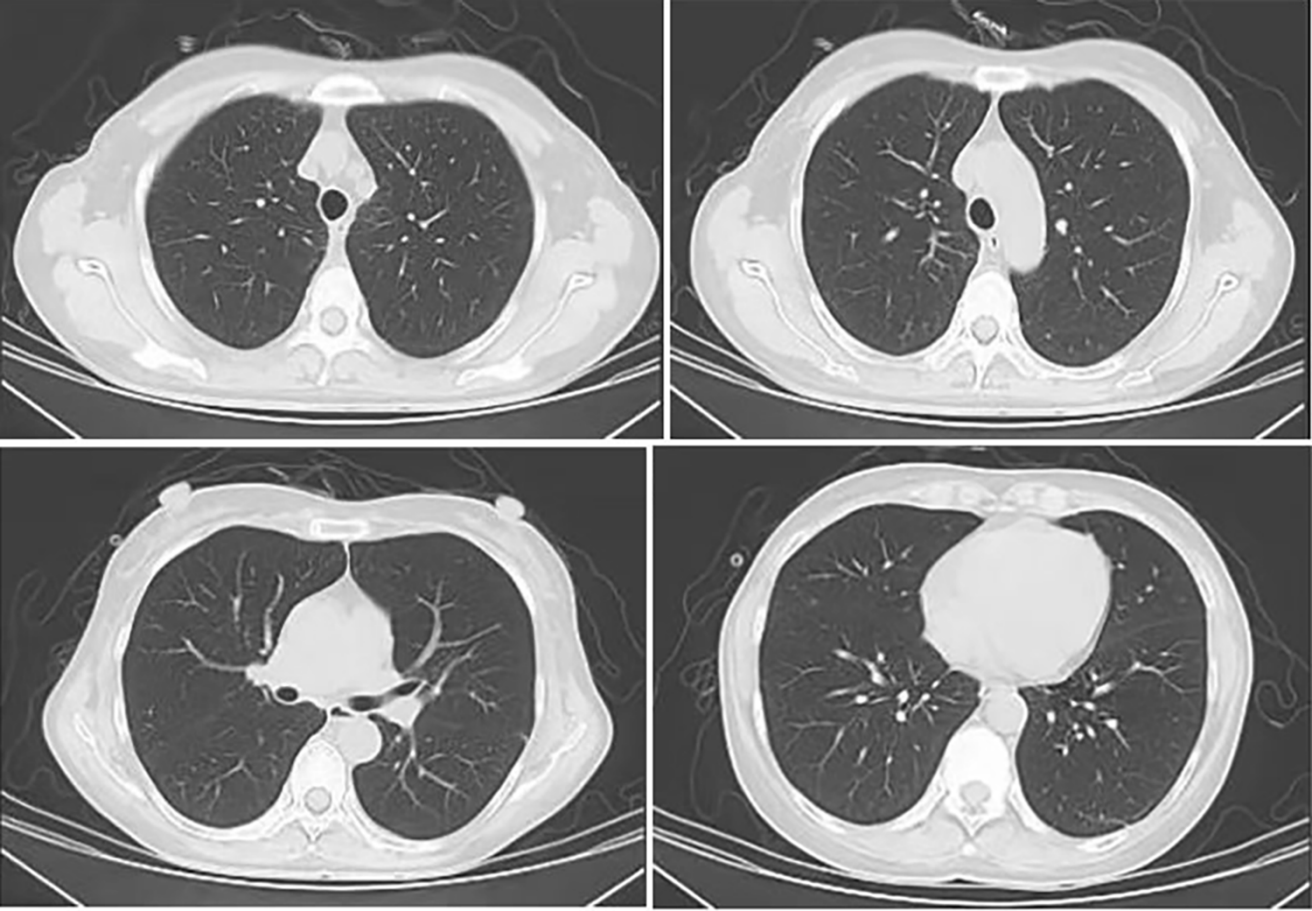
Chest computed tomography scans of immunocompetent patient in China who was co-infected with COVID-19 and non-HIV severe *Pneumocystis jirovecii* pneumonia, performed on July 17, 2023. All lung lesions were absorbed bilaterally.

On hospital day 8, antimicrobial treatment comprised oral sulfamethoxazole/trimethoprim (0.8 g/160 mg every 8 h), intravenous caspofungin (70 mg on the first day, then 50), and prednisone acetate (40 mg every 12 h for 5 days, 40 mg/d for 5 days, then 20 mg/d for 11 days). Concomitant treatments were intravenous human immunoglobulin, nutritional support, and noninvasive ventilator transnasal high-flow oxygen therapy. On day 2 after treatment, the patient’s fever abated; on day 3, shortness of breath resolved. On day 17, arterial blood gas indicated pH of 7.46, pCO_2_ 40 mm Hg, and pO_2_ 127 mm Hg (FiO_2_ 29%). Chest CT scan indicated that all lung lesions were absorbed bilaterally (Figure 4), and peripheral T-lymphocyte subsets comprised 561/μL CD4+ and 514/μL CD8+ T cells.

## Conclusions

Patients with COVID-19 who are older, have comorbidities, and are unvaccinated are susceptible to opportunistic infections such as aspergillus, candidiasis, and tuberculosis (*5*). Only a few cases have been reported in which immunosuppressed patients with moderate-to-severe COVID-19 pneumonia were co-infected with *P. jirovecii* (*5*). The prevalence of *P. jirovecii* infection in non-HIV patients (37 cases/1,000 patients) was significantly higher during the COVID-19 pandemic than before (13.1 cases/1,000 patients) (*6*). Moreover, before the pandemic, the mortality rate for non-HIV patients infected with PJP was higher (≈35%–50%) (*7*).

The prevalence of *P. jirovecii* colonization in immunocompetent persons varies. J.S. Kang found that 34.3% of patients with PJP had a history of COVID-19 and that 25.7% did not have underlying immunosuppressive conditions (*6*). The JiroCOVID study found that the prevalence of *P. jirovecii* colonization and infection was extremely low in immunocompetent patients, only 1.7% (*8*). Gentile et al. found that all but 1 patient with COVID-19 had no underlying immunosuppressive conditions or other risk factors for PJP infection (*9*). Thus, *P. jirovecii* infection is easily overlooked in patients without immunocompromise.

After patients are infected with COVID-19, the virus attacks the lymphocyte immune system, resulting in decreased CD4+ and CD8+ T-cell counts. The probability of opportunistic infection greatly increases when the CD4^+^ T-cell count is <200/μL (*3*). COVID-19 damages the epithelial barrier and induces an abnormal immune response resulting from immune system dysregulation (*6*). Unlike the patients reported by Gentile et al., who received treatment with corticosteroids for COVID-19 pneumonia, the patient we report had no other risk factors.

In China, only 4 patients with COVID-19 have been reported to be co-infected with PJP, including 2 patients with HIV infection and 2 older patients with chronic pulmonary diseases (*10*,*11*). COVID-19 co-infection with PJP has not been reported among patients who were immunocompetent and had no comorbidities. Although tests for COVID-19 returned negative results several times for the patient we report, we found SARS-CoV-2 RNA in BALF. Meanwhile, the peripheral T-cell count decreased to 80/μL, suggesting that the immune system was attacked by COVID-19. Therefore, for patients with atypical imaging manifestations, clinicians should search for opportunistic pathogens.

*P. jirovecii* is a fungus for which rates of colonization in the respiratory tract are low and is difficult to grow in vitro; therefore, it is difficult to detect through sputum cultures. β-d-glucan testing is considered an assistive method for diagnosis; sensitivity is 94% for HIV patients and 86% for non-HIV patients, and specificity is 83% for both groups of patients (*12*). Detection of *P. jirovecii* in BALF is considered the standard for PJP diagnosis.

Although *Candida* was detected in BALF from this patient, according to the 2016 guidelines of the Infectious Diseases Society of America, *Candida* isolated from the respiratory tract is primarily considered colonization (*13*). Therefore, we considered *Candida* colonization a possibility.

In the patient we report, the clinical manifestations of the disease in the early stages were atypical and could be easily misdiagnosed. Furthermore, SARS-CoV-2 tests conducted on nasopharyngeal swab samples and by sputum cultures yielded negative results. Fortunately, BALF testing using mNGS provided timely evidence for clinical diagnosis and treatment.

For patients with COVID-19 who are immunocompetent and not HIV infected but show persistently high fever and atypical viral or bacterial pneumonia on chest CT scan, clinicians should be highly vigilant of the possibility of PJP. Aggressive BALF and mNGS testing may help achieve a definitive diagnosis.
